# One-Time Acute Heat Treatment Is Effective for Attenuation of the Exaggerated Exercise Pressor Reflex in Rats With Femoral Artery Occlusion

**DOI:** 10.3389/fphys.2020.00942

**Published:** 2020-08-06

**Authors:** Lu Qin, Jianhua Li

**Affiliations:** Heart and Vascular Institute, Penn State University College of Medicine, Hershey, PA, United States

**Keywords:** femoral artery occlusion, muscle temperature, exercise, ATP, blood pressure

## Abstract

The purpose of this study was to determine the effects of one-time acute heat treatment (HT) on the exaggerated exercise pressor reflex in a model of peripheral arterial insufficiency induced by ligation of the femoral artery and was to further examine the underlying mechanism of ATP-P2X_3_ signal activity during this process. The blood pressure (BP) response to static muscle contraction and muscle tendon stretch was recorded to determine the exercise pressor reflex. Also, αβ-methylene ATP (αβ-me ATP) was injected into the arterial blood supply of the hindlimb muscles to stimulate P2X_3_ receptors in the muscle afferent nerves. To process one-time acute HT, a heating pad was placed locally on the hindlimb and the muscle temperature (Tm) was increased by ~1.5°C and maintained for 5 min. Compared with control rats, a greater mean arterial pressure (MAP) response to muscle contraction was observed in rats with femoral occlusion in a pre-heat control session (28 ± 2 mmHg in occluded rats/*n* = 12 vs. 18 ± 2 mmHg in control rats/*n* = 9; *p* < 0.05). The one-time acute HT attenuated the amplification of the BP response in rats with femoral artery occlusion (MAP response: 19 ± 8 mmHg in occluded rats + HT/*n* = 11; *p* < 0.05 vs. occluded rats). In contrast, HT did not significantly attenuate amplification of MAP response to muscle stretch and αβ-me ATP injection in rats with femoral artery occlusion and controls (all *p* > 0.05). Our data suggest that one-time acute HT selectively attenuates the amplified pressor response induced by activation of the metabolic and mechanical components of the reflex in rats after femoral artery occlusion. The suppressing effects of acute HT on the exaggerated exercise pressor reflex are likely mediated through a reduction in metabolites (e.g., ATP) stimulating the muscle afferent nerves in contracting muscle, but unlikely through direct alteration of P2X receptors *per se*.

## Introduction

On a global scale, peripheral artery disease (PAD) is one of the major cardiovascular concerns and affects more than 200 million individuals (Song et al., 2019). The clinical manifestation of intermittent claudication largely jeopardizes the daily mobility performance of PAD patients as it induces pain in legs during the physical activity (McDermott et al., 2004). Supervised exercise therapy has been long considered to be one of the first-line strategies for the treatment and life style management of PAD patients (Stewart et al., 2002; Hamburg and Balady, 2011). However, a risk of cardiovascular events should be considered for the patients during the exercise activity (Collins and Billman, 1989).

In the process of exercise, the mechanical stimuli and the metabolic products (e.g., lactate, ATP, and protons) generated in the contracting muscles activate the receptors in the Group III and IV muscle afferents (Li and Xing, 2012). The activation signals are transferred through the primary sensory dorsal root ganglion (DRG), the dorsal horn of spinal cord, and then projected to cardiovascular nuclei in the brainstem (Mitchell et al., 1983; Kaufman and Forster, 1996). The activation of the cardiovascular nuclei in the brainstem induces increases in the sympathetic nervous activity (SNA) and the consequent amplification of cardiovascular activities. The peripheral mechanism leading to increases in blood pressure (BP) and heart rate (HR) responses to muscle contraction during exercise was termed as the “exercise pressor reflex (EPR)” (Coote et al., 1971; McCloskey and Mitchell, 1972; Victor et al., 1988; Sinoway et al., 1989). The SNA also reduces muscle metabolite-mediated vasodilation (Shoemaker et al., 1999; Hammond et al., 2001), and therefore, limits the blood flow directed to the exercising muscles. The decreasing blood flow in the affected limb partly contributes to the exercise intolerance in PAD patients (Wilson and Mancini, 1993). Meanwhile, a greater increase in arterial BP, which is considered to be attributed to the exaggerated EPR, is more commonly observed in PAD patients than in normal subjects during walking (Baccelli et al., 1999; Bakke et al., 2007). Thus, it is important to study interventions and the underlying mechanisms to alleviate SNA and BP responses during exercise in PAD.

The ATP-P2X_3_ pathway has been considered to play a critical role in regulating EPR during the activation of primary sensory afferents in both healthy and cardiovascular disorders. Our previous studies have demonstrated that (1) compared with the resting status, the interstitial ATP concentration is enhanced when the muscle is active (Li et al., 2005), (2) the protein levels of the purinergic P2X_3_ expression are increased in the DRG of rats with 24–72 h of femoral artery occlusion (Liu et al., 2011), and (3) the BP response to stimulation of P2X_3_ by αβ-methylene ATP (αβ-me ATP; P2X_3_ agonist) in the afferent nerves is amplified in rats with simulated PAD (Liu et al., 2011; Xing et al., 2013). Meanwhile, it is noted that the functions of P2X receptors have also been reported to be altered by temperature in surrounding tissues (Garcia-Villalon et al., 1997; Ziganshin et al., 2002; Kluess et al., 2005). In a recent study, we demonstrated that the repeated heat exposure protocol [increasing ~1.5°C of muscle temperature (Tm) for 30 min, two times per day for three continuous days] effectively suppresses the P2X_3_ expression and attenuates the BP response to muscle contraction in rats with simulated PAD (Qin et al., 2020). Nonetheless, it is also necessary to determine a short-period of heating intervention relieving the symptoms of intermittent claudication.

Accordingly, following validating the effectiveness of repeated heating exposure on EPR in the previous study, in this report, we determined whether one-time acute heat treatment (HT) plays an inhibitory role in regulating BP response during activation of the exercise pressor reflex in rats with femoral artery occlusion. We hypothesized that one-time acute HT attenuates amplified BP response to muscle contraction in rats with femoral artery occlusion. We also hypothesized that the alterations in activities of ATP-P2X_3_ signal from contracting muscle to sensory nerves mediates this beneficial effect.

## Methods and Materials

### Animals

The animal experimental procedures were performed in compliance with the National Institutes of Health Guidelines and were approved by the Institutional Animal Care and Use Committee of the Penn State College of Medicine. Due to the potential effect of female sex hormones on the ATP-P2X_3_ pathway (Ma et al., 2011), only male Sprague-Dawley rats (250–350 g; Charles River Laboratory) were used in the present study. Animals were housed in individual cages with free access to standard food and in a temperature-controlled room (25°C) on a 12-h/12 h light/dark cycle.

### Animal Grouping

The ligation and sham surgery procedures were performed in two sets of rats. Those that underwent the right femoral artery occlusion served as “rats with femoral artery occlusion,” whereas those underwent sham surgeries on the right limb served as “control rats.” One-time acute HT was performed in some of the control rats and rats with femoral artery occlusion at 72 h after the surgery. Thus, in the present study four groups of animals were included: control, control + HT, rats with femoral artery occlusion, and rats with femoral artery occlusion + HT.

It is noted that a rat model with femoral artery occlusion does not fully exhibit all the clinical symptoms of PAD. It mimics the critical characteristics of insufficient blood flow, which is commonly observed in PAD (Waters et al., 2004). Thus, in this report, “PAD rats” was used to indicate peripheral arterial insufficiency but not to indicate that the rats had this disease.

### Femoral Artery Occlusion

After being anesthetized with an isoflurane-oxygen mixture (2–5% isoflurane in 100% oxygen), femoral artery ligation was performed as previously described (Lu et al., 2013; Xing et al., 2018). In brief, a ~1 cm surgical incision was made on the skin on right side of groin. After carefully cutting the fascia and removing the soft tissue around the veins, the femoral artery was exposed, dissected, and ligated with a surgical suture ~3 mm distal to the inguinal ligament. For the sham surgery, the same procedures were performed except that a suture was placed below the femoral artery without ligating the artery. Buprenorphine hydrochloride (0.05 mg/kg, subcutaneously) was administered prior to the surgery for post-operative pain relief. Following the surgery, the animals were kept in the surgery room for 2–3 h for observation, and then returned to the animal facility.

### Tm Monitoring and the One-Time Acute HT Procedure

Rats were anesthetized with an isoflurane-oxygen mixture (2–5% isoflurane in 100% oxygen) and placed on a surgery mat in the prone position. A temperature probe was carefully inserted into the middle point of the gastrocnemius for the continuous monitoring of limb Tm. To elevating the Tm, two heating pads were placed around the examined limb and were removed when the Tm increased by ~1.5°C. Thus, during HT, the Tm was maintained at ~1.5°C higher than the baseline. The length of the treatment process was 5 min.

### Surgical Preparations to Examine BP Response

The rats were anesthetized with a mixture of 2–5% isoflurane and oxygen and ventilated as described previously (Xing et al., 2018). The jugular vein and common carotid artery were cannulated. Fluids were delivered *via* the jugular vein while a pressure transducer (Model MLT0380, AD Instruments) was connected to the common carotid artery for measurement of the arterial BP *via* the Powerlab system. HR was calculated beat-to-beat from the arterial pressure pulse using the software of Lab Chart 7 (AD Instruments). To examine P2X-mediated cardiovascular responses, a catheter (PE10) was inserted into the femoral artery for injection of αβ-me ATP. In the rats with femoral artery occlusion, a small incision was made in the femoral artery distal to the previously occluded site. The catheter was then inserted into the artery toward the distal end to deliver the drug into the ischemic limb. During the experiments, baseline BP and fluid balance were maintained with a continuous infusion of saline. The body temperature was maintained at ~37°C with a temperature-controlled heat panel (TCAT-2LV, Physitemp Instruments, USA).

Decerebration was performed to eliminate the effects of anesthesia on the reflex pressor response. Prior to the procedure, dexamethasone (0.2 mg, i.v.) was injected to minimize brain stem edema. After decerebration, anesthesia was withdrawn from the rats, and the animals were switched to a ventilator (Model 683, Harvard Apparatus, USA).

A laminectomy procedure (Smith et al., 2001) was performed to expose the lower lumbar and upper sacral portions of the spinal cord and the peripheral ends of the transected L4 and L5 ventral roots were placed on platinum bipolar stimulating electrodes. Experiments were performed 60 min later. Static muscle contractions were induced by electrical stimulation of the L4 and L5 ventral roots (30 s, 3 times motor threshold with a period of 0.1 ms at 40 Hz). In order to activate the mechanical component of the exercise pressor reflex, passive tendon stretch was performed in the right hindlimb. Passive tendon stretch (500 g of tension) was produced manually over ∼5 s by using a rack and pinion attached to the Achilles’ tendon of rats. Each bout of muscle stretch was maintained for 30 s after 500 g of tension was achieved.

### Examination of the EPR

The BP and HR responses to muscle contraction and tendon stretch were examined in control, control + HT, PAD, and PAD + HT. The reflex responses were assessed in three sessions including: 20 min before the one-time acute HT (control session, CON), immediately following the HT (HT session), and 20 min after the HT (recovery session, REC).

In additional groups, BP response to arterial injections of αβ-me ATP (0.0625 mM, 0.125 mM, 0.4 ml/kg) was examined in the four groups of rats. The concentrations were selected according to our published work (Liu et al., 2011). The duration of the injection was 1 min and an interval of 20 min was allowed between injections. At the end of the experiments, the animals were euthanized by inhalation of an overdose of isoflurane followed by cardiac puncture.

### Statistical Analysis

Unless specified, the data in this study are presented as the mean ± SD. The SPSS for Windows version 26.0 was utilized for all statistical analyses. Two-way ANOVA was applied to compare the differences in BP and HR responses among pre-heat CON, HT, and REC sessions in control rats and PAD rats. As appropriate, *post hoc* analysis with Tukey’s test was applied to compare the differences between specific groups. A *p* less than 0.05 was considered as statistical significance.

## Results

### Tm in the Hindlimb Muscle

**Figure 1:**
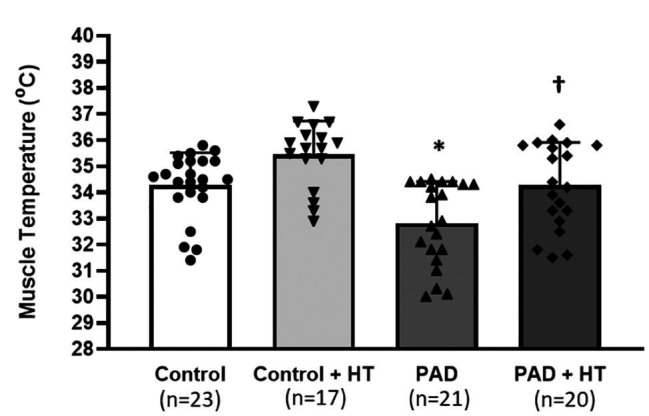
Basal muscle temperature and muscle temperature following the one-time acute heat treatment (HT) in control rats and peripheral artery disease (PAD) rats. Baseline muscle temperature was lower in PAD rats (*n* = 21) than that in control rats (*n* = 23). Following the HT, the muscle temperature was increased by ~1.5°C. As a result, the muscle temperature was also lower in PAD + HT rats (*n* = 20) than the control + HT rats (*n* = 17). The time length for HT was 5 min. ^*^ *p* < 0.05 for baseline muscle temperature between control rats and PAD rats; ^†^ *p* < 0.05 for the muscle temperature following the HT between control + HT rats and PAD + HT rats. HT = one-time acute heat treatment. The numbers in the bracket under each group indicate the sample size. Individual data points are indicated for Control group (●); Control + HT group (▼); PAD group (▲); and PAD + HT group (♦).

The baseline Tm in control group was significantly higher than that in the PAD group. They were 34.26 ± 1.37°C in control rats vs. 32.81 ± 1.38°C in PAD rats (*p* < 0.05, between control and PAD). With the protocol of HT, the Tm was 35.45 ± 1.26°C in control rats vs. 34.27 ± 1.39°C in PAD rats (*p* < 0.05, between control + HT and PAD + HT). Note that the Tm for control rats was ~1.5°C higher than that in PAD rats either before or after HT in this report (Figure 1).

### Blood Pressure Response Following Static Muscle Contraction

**Figure 2:**
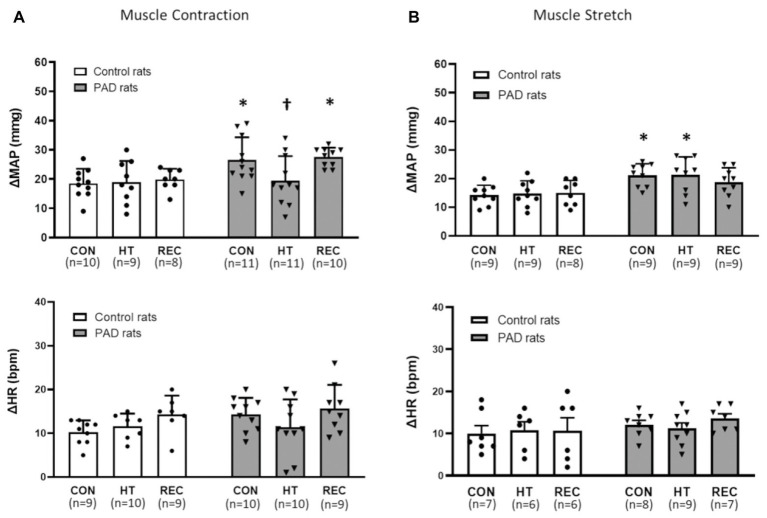
Responses of mean arterial blood (MAP) pressure and heart rate (HR) in control rats and PAD rats induced by muscle contraction and muscle stretch. In control (CON) and recovery (REC) session, MAP response to muscle contraction **(A)** was amplified in PAD rats when compared with the control rats. In HT session, the MAP response to muscle contraction in PAD rats was lower than that in CON; MAP response to muscle stretch **(B)** were higher in PAD rats than in control during either CON and HT session; ^*^ *p* < 0.05 vs. control rats and ^†^ *p* < 0.05 vs. PAD rats in the control session. There were no significant differences in the HR response during the muscle contraction **(A)** and muscle stretch **(B)** in different groups and sessions (*p* > 0.05). CON = pre-heat control session; HT = one-time acute heat treatment; and REC = post-heat recovery session. The numbers in the bracket under each session name indicate the sample size. ●: individual data points for Control rats; ▼: individual data points for PAD rats.

No significant differences in basal MAP were found among different heating sessions in the control and PAD groups (*p* > 0.05; Table 1). A greater MAP response following static muscle contraction was observed in PAD rats (26 ± 8 mmHg in PAD rats/*n* = 11 vs. 18 ± 5 mmHg in control rats/*n* = 10; *p* < 0.05) during the pre-heat CON session. The one-time acute HT attenuated the amplification of the BP response in PAD rats (MAP response: 19 ± 8 mmHg in PAD rats + HT/*n* = 11; *p* < 0.05 vs. PAD rats). In post-heat REC session (20 min after the HT), a greater MAP response during static muscle contraction was regained in PAD rats (27 ± 3 mmHg in PAD rats/*n* = 10 vs. 20 ± 4 mmHg in control rats/*n* = 8; *p* < 0.05). Note that HT did not significantly attenuate the MAP response to muscle contraction in control animals (18 ± 7 in control + HT vs. 18 ± 5 mmHg in control; *p* > 0.05 between two groups; Figure 2A). There were insignificant differences in the HR response and developed muscle tension during static contraction in different groups and sessions (*p* > 0.05; Figure 2A; Table 2).

**Table 1:** The baseline values for blood pressure (BP) in muscle contraction and muscle stretch experiments.

		CON		HT		REC	
		Control rats	PAD rats	Control rats	PAD rats	Control rats	PAD rats
Muscle contraction	Sample size (*N*)	10	11	9	11	8	10
	BP (mmHg)	91 ± 14	93 ± 14	95 ± 16	95 ± 14	90 ± 6	96 ± 13
Muscle stretch	Sample size (*N*)	9	9	9	9	8	9
	BP (mmHg)	88 ± 12	92 ± 17	86 ± 13	104 ± 20	89 ± 14	98 ± 20

BP, blood pressure; CON, pre-heat control session; HT, one-time acute heat treatment; and REC, post-heat recovery session. No significant differences in basal MAP before contraction and stretch were found among different sessions in the two groups and two different groups in the same session (*p* > 0.05). All the data were present as mean ± SD.

**Table 2:** Developed muscle tension during contraction in different groups and sessions.

	CON		HT		REC	
	Control rats	PAD rats	Control rats	PAD rats	Control rats	PAD rats
Sample size (*N*)	9	11	8	11	7	10
Tension (g)	557 ± 104	485 ± 105	501 ± 84	560 ± 130	604 ± 104	575 ± 75

CON, pre-heat control session; HT, one-time acute heat treatment; REC, post-heat recovery session; and g, gram. No significant differences in muscle tension among different sessions in the two groups and two different groups in the same session (*p* > 0.05). All the data were present as mean ± SD.

### Blood Pressure Response Following Muscle Stretch

No significant differences in basal MAP were found among different heating sessions in the control and PAD groups (*p* > 0.05; Table 3). Compared with the control rats, greater MAP responses during muscle tendon stretch were observed in PAD rats during pre-heat CON and HT sessions (pre-heat control session: 21 ± 4 mmHg in PAD rats/*n* = 9 vs. 14 ± 3 mmHg in control rats/*n* = 9; HT session: 21 ± 6 mmHg in PAD rats/*n* = 9 vs. 15 ± 4 mmHg in control rats/*n* = 9; *p* < 0.05 between two groups during two sessions). Likewise, HT had no significant inhibitory effects on the MAP response to muscle stretch in both control and PAD rats, as no significant difference was found in both groups among difference sessions (CON, HT, and REC; *p* > 0.05). In addition, there were no significant differences in the HR response during muscle stretch in different groups and sessions (*p* > 0.05; Figure 2B).

**Table 3:** The baseline values for BP in 0.0625 and 0.125 mM αβ-me ATP administration experiments.

		CON		HT		REC	
		Control rats	PAD rats	Control rats	PAD rats	Control rats	PAD rats
0.0625 mM	Sample size (*N*)	5	9	5	9	4	8
	BP (mmHg)	96 ± 23	99 ± 21	93 ± 25	95 ± 20	99 ± 24	93 ± 16
0.125 mM	Sample size (*N*)	8	7	8	7	7	6
	BP (mmHg)	93 ± 17	90 ± 20	89 ± 35	90 ± 14	91 ± 14	88 ± 6

BP, blood pressure; CON, pre-heat control session; HT, one-time acute heat treatment; and REC, post-heat recovery session. No significant differences in basal MAP before injection of each dosage of αβ-me ATP were found among different sessions in the two groups and two different groups in the same session (*p* > 0.05). All the data were present as mean ± SD.

### Blood Pressure Response Following αβ-me ATP Injection

**Figure 3:**
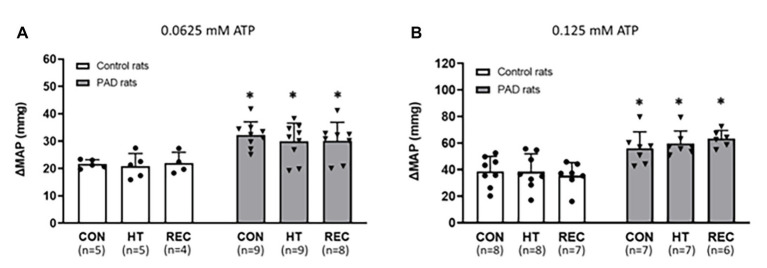
Responses of MAP in control rats and PAD rats following 0.0625 mM **(A)** and 0.125 mM **(B)** of αβ-methylene ATP (αβ-me ATP) administration. In three sessions of CON, HT and REC, MAP response to 0.0625 mM and 0.125 mM of αβ-me ATP administration were higher in PAD rats than in control rats. However, the MAP response was not attenuated in either PAD and in control rats following the one-time acute HT. ^*^ *p* < 0.05 vs. control rats. CON = pre-heat control session; HT = one-time acute heat treatment; and REC = post-heat recovery session. The numbers in the bracket under each session name indicate the sample size. ●: individual data points for Control rats; ▼: individual data points for PAD rats.

There were no significant difference in the baseline values of MAP among the different heating sessions in control rats and PAD rats (*p* > 0.05 between two groups for two dosages of αβ-me ATP; Figure 3; Table 3). Following 0.0625 and 0.125 mM of αβ-me ATP injection, the MAP response in PAD rats was significantly higher than that in control rats during the CON session [0.0625 mM: 32 ± 5 mmHg in PAD (*n* = 9), vs. 22 ± 2 mmHg in control rats (*n* = 5), *p* < 0.05 and 0.125 mM: 57 ± 8 mmHg in PAD rats (*n* = 7) vs. 39 ± 5 mmHg in control rats (*n* = 8), *p* < 0.05]. The greater MAP response to αβ-me ATP remained during HT and REC session. During HT session, the MAP responses induced by 0.0625 mM of αβ-me ATP were 30 ± 7 mmHg in PAD (*n* = 9) vs. 21 ± 5 mmHg in control (*n* = 5; *p* < 0.05 vs. PAD) and induced by 0.125 mM of αβ-me ATP were 61 ± 6 mmHg in PAD rats (*n* = 7) vs. 40 ± 5 mmHg in control rats (*n* = 8; *p* < 0.05 vs. PAD). During REC session, the MAP response induced by 0.0625 mM of αβ-me ATP was 30 ± 7 mmHg in PAD (*n* = 8) vs. 20.93 ± 4.55 mmHg in control rats (*n* = 4; *p* < 0.05) and induced by 0.125 mM of αβ-me ATP was 61 ± 6 mmHg in PAD rats (*n* = 6) vs. 40 ± 5 mmHg in control rats (*n* = 7; *p* < 0.05). This figure also shows that HT did not attenuate the MAP response to both dosages of αβ-me ATP injection in control rats (*p* > 0.05; Figures 3A,B).

## Discussion

In the present study, we demonstrated that the one-time acute HT attenuated the BP response to muscle contraction in rats with femoral artery occlusion (Figure 2A), but had no significant effects on the reflex BP response to either the passive muscle stretch or the αβ-me ATP injection (Figures 2B, 3A,B).

The role of ATP-P2X_3_ pathway in the regulation of EPR in PAD has been studied previously (Li et al., 2005; Liu et al., 2011; Xing et al., 2013). During muscle contraction, ATP utilization is accelerated to meet the energy demand. Under ischemia, the production of reactive oxidative species (ROS) inhibits ATPase activity in the mitochondria, and therefore, reduces intracellular ATP utilization (Dobrota et al., 1999; Simao et al., 2011; Jeong et al., 2013). Apart from the suppressed intracellular ATP utilization, during the ischemia condition, the muscular cells are swollen, and thus, the permeability and efflux of the cytoplasmic soluble molecules including ATP is increased (Boudreault and Grygorczyk, 2002). With the increasing amount of intracellular ATP and the enhanced permeability of the ischemic cell membrane, it can be deduced that the interstitial ATP is enhanced during the muscle ischemia and elicits a greater stimulation on the P2X receptors (receptors for ATP) in the muscle afferent nerves. Among the P2X receptors, P2X_3_ subtype, specifically, is presented in the primary sensory neurons in DRG (Chen et al., 1995). The elevated ATP concentration in the muscle interstitium enhances the expression and function of P2X_3_ and the subsequent EPR response is exaggerated. Consistent with previous studies (Liu et al., 2011; Qin et al., 2020), the BP response following both muscle contraction and muscle stretch was higher in rats with femoral artery occlusion than that in control rats (Figures 2A,B). BP response to stimulation of P2X_3_ by injection of αβ-me ATP, as a part of EPR activation, was also amplified in PAD rats (Figures 3A,B).

It has been suggested that an exaggerated SNA response to exercise in cardiovascular diseases lowers the ventricular fibrillation threshold, and therefore, increases the probability of cardiovascular risk (e.g., fatal arrhythmias) in patients (Collins and Billman, 1989; Vanoli and Schwartz, 1990). HT, as an inexpensive and non-invasive intervention strategy, has been considered as one of the promising interventions to attenuate the BP response in the PAD patients. It was reported in an early study that, by elevating the intramuscular temperature from ~32.2 to ~41.5°C, action potential amplitude in the sensory nerves was significantly reduced by 32–84% and the duration reduced by 23–36% (Rutkove et al., 1997). This result suggested that there might be a negative association between the muscular temperature and the activity of the primary muscle afferent nerves during static exercise. To date, there are a few clinical studies to investigate one-time acute effect of HT on the BP response in PAD patients. For instance, a previous study by Neff et al. (2016) revealed that increasing leg skin temperature to 39–40°C for 90 min significantly attenuated both systolic and diastolic BP in PAD patients with intermittent claudication. Immersion of the lower limbs in water at 42°C for 30 min also decreased arterial BP in PAD patients (Thomas et al., 2017). With the approach of ventral root stimulation in the current study, it was observed that the BP response to the skeletal muscle contraction was enhanced in PAD rats when compared with the control rats. This result was also consistent with what observed in the previous studies (Liu et al., 2011; Qin et al., 2020). In addition, the exaggerated BP response was attenuated following increasing Tm by ~1.5°C (Figure 2A).

One explanation for the HT-attenuated BP response following the muscle contraction is improvement of suppressed ATPase activity in the skeletal muscle of PAD rats. It has been reported that the change of tissue temperature alters cellular enzyme activity. For instance, intrinsic ATPase activity (Paudel and Carlson, 1991) in the New Zealand rabbit skeletal muscle is gradually increased from 0 to 30°C (one-fold increase) and dramatically accelerated from 30 to 40°C (approximately six-fold increase). In contrast, in our present study (Figure 1), the average baseline Tm was 34.26 ± 1.37°C in control rats and 32.81 ± 1.38°C in PAD rats. After the HT, the average terminal Tm was 35.45 ± 1.26°C in control + HT rats and 34.27 ± 1.39°C in PAD + HT rats. These temperatures were within the range from 30 to 40°C. This suggests that, within that range, the Tm increment of ~1.5°C likely amplified the activity of ATPase in the skeletal muscle in the current study. Moreover, a human study showed that the elevation of Tm was associated with increased ATP turnover rates during the exercise (Gray et al., 2006).

The inhibitory effects of HT on the BP response induced by passive muscle stretch were not observed in PAD rats. It could be hypothesized that muscle stretch stimulated only the mechano-receptors in the muscle afferent nerves, and a short session of HT was unlikely to affect the process of mechanical activation. However, muscle contraction induces an increase in muscle metabolic products and thereby stimulates on both the metabo- and mechano-receptors in muscle afferents in evoking the reflex BP response (Li and Xing, 2012). As a result, there was likely less ATP released into the interstitial space of muscle during the muscle contraction in PAD rats following this short-time HT protocol. Therefore, there was also a smaller subsequent BP response seen in the current study. In addition, the attenuating effects of 5 min of HT on the BP response to muscle contraction were abolished when the Tm was recovered during the recovery session. This suggests that the BP attenuation was a temporary process since the Tm was not elevated during the recovery session.

In our previous study (Qin et al., 2020), using a repeated heating exposure protocol, overexpression of P2X_3_ protein in the DRG was attenuated in PAD rats following three-day heating treatment. As a consequence, the BP response to αβ-me ATP injection in the arterial blood supply of the hindlimb muscles was attenuated following that heating protocol. In contrast, 5 min of HT did not attenuate the exaggerated BP response to arterial injection of αβ-me ATP in rats with femoral artery occlusion (Figures 3A,B). This suggests that this short HT protocol did not alter protein expression of P2X_3_, and thus, the amplitude of P2X_3_ response stimulated by the αβ-me ATP was unchanged following the HT. In a previous study (Tsuzuki et al., 2001), P2X_3_ messenger RNA (mRNA) expression was examined following peripheral nerve injury, demonstrating that P2X_3_ mRNA in the DRG was enhanced from day 1 post-injury. Our previous study (Liu et al., 2011) also indicated that the P2X_3_ expression in the DRG was not upregulated at 6 h but started to increase 24 h following the femoral artery ligation. Likewise, the attenuation of the exaggerated P2X_3_ expression in the DRG of PAD rats also requires a time course, as shown that there was 3 h of accumulated heating exposure in the previous repeated heating protocol (Qin et al., 2020). Moreover, during the repeated heating treatment, half of the heating procedures were already completed during the first 24 h following the ligation, which was before P2X_3_ upregulation. In contrast, 5 min of HT was unlikely to attenuate the protein P2X_3_ overexpression in rats with femoral artery occlusion, which explains why 5 min of HT failed to attenuate BP response to α,β-me-ATP. However, a likelihood cannot be ruled out that HT could alter the sensitivity of P2X_3_ receptors on the peripheral endings of afferents, and thus suggests involvement of the reflex BP response. In addition, without contraction the intracellular ATP failed to efflux into the interstitial space of resting muscle during αβ-me ATP injection. Therefore, rather than stimulated by the ATP released into the interstitial space by muscle contraction, the P2X_3_ in the primary sensory afferent were stimulated by injection of αβ-me ATP *per se*. This somewhat explains why in the current study the higher BP response to αβ-me ATP injection remained in rats with femoral artery occlusion following HT.

In the present study, acute HT attenuated the BP response to static contraction, but it did not attenuate the BP response to respective activation of muscle mechnoreflex and P2X_3_ receptors. It seems that the role of metabolites as a mechanism for HT to change BP response to contraction cannot be explained by results of the αβ me ATP injection. Thus, the metabolic component of the exercise pressor reflex was responsible for the attenuation of the BP response to static contraction following acute HT cannot be evidently concluded until some other metabolites are identified in engagement of acute HT. On the other hand, muscle contraction led to the release of ATP into the interstitial space. With the enhancement of intracellular ATPase activity following the HT, the amount of ATP released into the interstitial space during muscle contraction was likely less, and therefore, the subsequent BP response was diminished.

In conclusion, one-time acute HT attenuates the amplified pressor response induced by static muscle contraction in rats with femoral artery occlusion, but not by muscle stretch and by stimulation of P2X in muscle afferents. The suppressing effects of one-time acute elevation of Tm on the exaggerated exercise pressor reflex in PAD are likely through reducing the activity of temperature sensitive muscle metabolites (e.g., ATP, lactate, and proton) in contracting muscle thereby leading to attenuation of the reflex. Nonetheless, P2X expression and/or responsiveness in muscle sensory nerves might not be altered during the one-time acute HT *per se*. Whether or not the sensitivity of P2X receptors on the peripheral endings of afferents is altered during HT and thereby affecting the exercise pressor reflex in PAD needs to be determined.

## Perspective

In the present study, static muscle contraction and muscle tendon stretch were used to evoke the exercise pressor reflex in a rat model of femoral artery occlusion. Of note, static exercise protocols are not surrogates for dynamic exercise in humans or treadmill exercise in rats. Therefore, caution should be taken when extrapolating the findings of the current study to whole-body dynamic exercise. The neural mechanisms involved in the effects of acute HT on the exercise pressor reflex still need to be examined using the dynamic exercise models. Additionally, future studies will need to investigate the effects of acute HT on the exercise pressor reflex in females.

The experiments were conducted 3 days following femoral artery occlusion. It could be argued that HT should be tested when the natural compensation to arterial occlusion has stabilized, in part, because the mechanisms mediating initial responses to acute occlusion may differ from those involved in later stages of adaptation (Ziegler et al., 2010). Indeed, the muscle metabolic and inflammatory milieu are largely disturbed in the first few days after the occlusion, creating a scenario that does not mimic the human condition. The physiological responses to exercise in this model are likely exacerbated in the first few days after femoral artery ligation. That is, Prior et al. (2004) reported that in rats BP response during treadmill exercise was greater within 2 days after femoral artery ligation than that after 25 days. It is therefore important to recognize that the reported effects of acute heat treatment may be different, if the experiments are conducted when natural compensation has stabilized.

## Data Availability Statement

All datasets presented in this study are included in the article/supplementary material.

## Ethics Statement

The animal protocol was reviewed and approved by the Institutional Animal Care and Use Committee of the Penn State University College of Medicine.

## Author Contributions

QL processed cardiovascular response experiments for the drug administration, contributed to the analysis of cardiovascular recording, and drafted the manuscript. LJ designed experiments, oversaw performance of the experiments and data analysis, and revised the manuscript. All authors approved the final version of the article submitted for publication.

### Conflict of Interest

The authors declare that the research was conducted in the absence of any commercial or financial relationships that could be construed as a potential conflict of interest.
